# β-sheets in serum protein are independent risk factors for coronary lesions besides LDL-C in coronary heart disease patients

**DOI:** 10.3389/fcvm.2022.911358

**Published:** 2022-08-09

**Authors:** Yu-lin Li, Jia-ying Xie, Bin Lu, Xiao-di Sun, Fang-fang Chen, Zhou-jie Tong, Wen-wen Sai, Wei Zhang, Zhi-hao Wang, Ming Zhong

**Affiliations:** ^1^The Key Laboratory of Cardiovascular Remodeling and Function Research, Chinese Ministry of Education, Chinese National Health Commission and Chinese Academy of Medical Sciences, The State and Shandong Province Joint Key Laboratory of Translational Cardiovascular Medicine, Department of Cardiology, Qilu Hospital, Cheeloo College of Medicine, Shandong University, Jinan, China; ^2^Department of Geriatric Medicine, The Second Hospital, Cheeloo College of Medicine, Shandong University, Jinan, China; ^3^Department of Geriatric Medicine, Qilu Hospital, Cheeloo College of Medicine, Shandong University, Shandong Key Laboratory of Cardiovascular Proteomics, Jinan, China

**Keywords:** β-sheet, coronary heart disease, LDL-C, atherosclerosis, misfolded proteins

## Abstract

**Background:**

Coronary heart disease (CHD) patients with standard low-density lipoprotein cholesterol (LDL-C) remain at risk of cardiovascular events, making it critical to explore new targets to reduce the residual risk. The relationship between β-sheet conformation and CHD is gaining attention. This study was designed to compare the coronary lesions in CHD patients with varying LDL-C and evaluate whether serum β-sheets are associated with coronary damage.

**Methods:**

Two hundred and one patients diagnosed with stable CHD were recruited and divided into four groups according to LDL-C. Baseline information, coronary lesion-related indicators, and peripheral blood samples were collected. Serum β-sheet content was determined by thioflavin T fluorescence.

**Results:**

The baseline information was comparable in CHD patients with different LDL-C. No difference was found in indicators relevant to coronary lesions among groups. The content of β-sheet was negatively correlated with LDL-C. Multiple linear regression revealed that serum β-sheet was positively correlated with coronary lesion when risk factors such as age, smoking, and LDL-C were controlled.

**Conclusions:**

This is the first study that reports the serum β-sheet levels of CHD patients being gradually increased with decreasing LDL-C when coronary lesions were comparable. Serum β-sheet might exacerbate the coronary lesions in CHD patients independent of known risk factors such as LDL-C.

## Introduction

The burden of coronary heart disease (CHD) has risen to become a dominant public health problem and a top cause of mortality worldwide in recent years (1). CHD is demonstrated to be the second leading cause of death (2) and the second major cause of premature death (3) in the Chinese population. CHD is exacerbated by increased low-density lipoprotein cholesterol (LDL-C). Therefore, LDL-C is recommended as the primary intervention target for CHD by various guidelines (4). Nevertheless, CHD patients still have a high residual risk of cardiovascular events even with the optimal treatment and State-of-the-Art secondary prevention therapies (5, 6). It is probable that factors besides LDL-C, such as dyslipidemia characterized by high triglyceride (TG) and low high-density lipoprotein cholesterol (HDL-C) (7, 8) are involved in atherosclerosis. Then, it is critical to find unknown factors except for LDL-C for the intervention of coronary lesions.

Lipid deposition in the intima is a key link to atherosclerosis (9). Current research has been focusing on the involvement of cholesterol rather than apolipoprotein (Apo) in coronary lesions. It is controversial whether the apolipoprotein is involved in atherosclerosis. The deposition of ApoA-I, ApoB, ApoC-II, and ApoE has been revealed by immunohistochemistry in human coronary plaques (10). Furthermore, an obvious increase in β-sheet and a decline in α-helix in the secondary structure of proteins have been found in the plaques of atherosclerotic mice by Fourier transform infrared microspectroscopy imaging (11). A fitting analysis of the amino acid sequences suggests that the α2 domain of ApoB, a structural protein of LDL-C, was easily transformed from α-helix to β-sheet during LDL-C oxidation (12). Circular dichroism analysis also indicates that oxidized LDL-C (ox-LDL-C) showed a significant decline in α-helical structure compared with native LDL-C (33.5% vs. 93.2%), whereas the content of β-sheet was increased (16.4% vs. 6.2%) (13), resulting in the increase of β-sheet content in plaques. X-ray powder diffraction patterns have confirmed that the abundant β-sheet of ApoB in atherosclerosis had the same pathogenic antiparallel structure as Aβ in Alzheimer's disease (14). In addition, β-sheet-rich misfolded proteins are prone to form amyloid fibers, which is associated with the conversion of macrophages to a pro-inflammatory phenotype, the increment of reactive oxygen species, and the inflammatory response of atherosclerosis (15, 16). In summary, elevated β-sheet in oxidatively modified LDL-C is likely to be a major source of misfolded proteins in atherosclerotic plaques. Abundant β-sheet in serum protein, especially apolipoprotein, not only reflects oxidative modification of LDL-C but may also promote the development of atherosclerosis.

In this study, we compared the differences in coronary lesions in CHD patients with varying LDL-C levels. Afterward, we investigated the relationship between serum β-sheet levels and coronary damage.

## Materials and methods

### Subjects

The participants in this study were 201 stable coronary heart disease patients admitted to Qilu Hospital of Shandong University from June 2018 to June 2019. According to the level of LDL-C, patients were divided into four or three groups. The primary inclusion criteria for the participants was one or more coronary artery stenosis ≥ 50%, according to coronary angiography results. Exclusion criteria included acute coronary syndrome (cardiac troponin I < 17 ng/L), coagulation disorders, active bleeding, cerebrovascular accidents, acute and chronic infectious diseases, malignant tumors, immune system diseases, severe liver and kidney abnormalities, and abnormal psychiatric and mental functions that prevent normal communication. All patients provided written informed consent and the Ethics Committee of the Qilu Hospital of Shandong University approved this study. We strictly followed the ethical guidelines of the declaration of Helsinki.

### Basic data collection and gensini score calculation

Basic clinical data collected included information from physical examination, medical history, lab examination, and medical therapy. The severity of coronary artery lesions was determined from the patient's coronary angiography and coronary imaging data. Four indicators were collected, namely, the degree of stenosis (expressed as a percentage) of each coronary lesion, the number of segments of stenotic lesions, the number of arteries involved in the coronary lesions and the specific arteries, and the Gensini score.

Gensini score was calculated according to the severity of stenosis and coronary artery position (17). Obstruction of 25, 50, 75, 90, 99, and 100% were given a score of 1, 2, 4, 8, 16, and 32, respectively. This score was then multiplied by a factor that took into account the importance of the lesion position in the coronary arterial tree. Specifically, in patients with the right dominant coronary system, 5 for the left main coronary artery, 2.5 for the proximal segment of the left circumflex artery (LCX) or proximal segment of the left anterior descending artery (LAD), 1.5 for the mid-segment of LAD, 1 for the mid and distal segment of LCX, obtuse marginal, an apical segment of LAD, first diagonal, right coronary artery or posterior descending artery, and 0.5 for any other branches. In patients with the left dominant coronary system, the multiplying factor of proximal, mid, and distal segments of LCX is 2, 2, and 3.5 respectively, and other segments are consistent with the right dominant coronary system. The final score is the sum of all the lesion scores.

All assessments were carried out by the same technician. These indicators are used to assess the severity of coronary artery disease in a comprehensive manner.

### Thioflavin T (ThT) staining

Typically, the β-sheet structure is separated by the α-helix in protein. The α-helix is converted into a β-sheet when the folding method of the peptide chain is changed (12). Due to the absence of α-helical blocking, β-sheet spontaneously replicates and forms pathogenic amyloid specifically labeled by ThT dye (18). Therefore, ThT staining was utilized to detect serum β-sheet levels.

Four-milliliter venous blood samples were collected following overnight fasting for 12–14 h. Serum was separated by centrifugation at 1,000 *g* for 20 min and placed in −80°C cryopreservation. ThT fluorescence was assayed using procedures described previously by several authors (19–21). We prepared 6 mM ThT solutions and set the spectrophotometer parameters: excitation wavelength 440 nm, slot 2.5 nm; emission wavelength 485 nm, slot 5 nm. A total of 5 μl of serum was added with 240 μl of PBS (pH 7.4) and mixed. Then 5 μl of ThT solution was added. The system was incubated at room temperature and protected from light for 20 min. The fluorescence intensity was monitored by the Cary Eclipse Fluorescence Spectrophotometer (Varian, Palo Alto, United States). Under these conditions, the intrinsic fluorescence of serum was negligible. Three replicate wells (parallel samples) were set for each sample and the average value was taken as the fluorescence intensity of the sample.

### Statistical analyses

All statistical analyses were carried out using SPSS (version 25.0, SPSS, Chicago, IL, United States). Data transformation was accomplished, and all data met with the normal distribution. Enumeration data were expressed by the number of cases and the constituent ratio [*n* (%)], and the chi-square test was used for comparison. Measurement data were expressed as mean ± SD, and a comparison between four groups was analyzed by one-factor analysis of variance. In case of a significant difference, the LSD *t*-tests were performed to compare the significant difference between the two groups. Multivariate linear regression analyses were used to explore the influencing factors of the Gensini score. Statistical significance was taken at *P* < 0.05.

## Results

### Baseline characteristics

Of the 201 patients enrolled in the trial, there were 22 individuals with LDL-C ≤ 1.4 mmol/L, 30 with 1.4 mmol/L < LDL-C ≤ 1.8 mmol/L, 84 with 1.8 mmol/L < LDL-C ≤ 2.6 mmol/L, and 65 with LDL-C > 2.6 mmol/L. As shown in Table 1, age and physical examination (systolic blood pressure, diastolic blood pressure, body mass index, and waist-to-hip ratio) in four groups were comparable (*P* > 0.05). In terms of medical history, participants had comparable smoking status, the condition of hypertension, diabetes and previously diagnosed myocardial infarction, as well as a family history of diabetes, and CHD (*P* > 0.05, Table 1). In laboratory examination, the glycosylated hemoglobin, fasting blood glucose, albumin (ALB), prealbumin (PA), and homocysteine level of participants were comparable (*P* > 0.05, Table 1).

**Table 1:** Baseline characteristics of CHD patients with different LDL-C levels.

**Characteristic**	**LDL-C ≤ 1.4 mmol/L (*n* = 22)**	**1.4 mmol/L < LDL-C ≤ 1.8 mmol/L (*n* = 30)**	**1.8 mmol/L < LDL-C ≤ 2.6 mmol/L (*n* = 84)**	**LDL-C > 2.6 mmol/L (*n* = 65)**	***P***
Age (y.o)	58.59 ± 10.08	62.09 ± 8.08	61.84 ± 8.61	61.46 ± 10.04	0.491
Gender [*male* (%)]	14 (63.6%)	19 (63.3%)	49 (58.3%)	39 (60%)	0.950
**Physical examination**					
Systolic blood pressure (mmHg)	131.23 ± 17.90	137.12 ± 20.81	135.92 ± 17.09	139.61 ± 18.01	0.279
Diastolic blood pressure (mmHg)	76.68 ± 13.27	74.33 ± 12.88	76.60 ± 12.24	77.48 ± 10.72	0.700
BMI (Kg/m^2^)	25.83 ± 3.25	26.43 ± 3.06	25.44 ± 3.67	26.11 ± 3.10	0.471
Waist-to-hip ratio	0.96 ± 0.05	0.96 ± 0.06	0.94 ± 0.06	0.94 ± 0.06	0.222
**Medical history**					
Smoking [yes (%)]	12 (54.5)	17 (56.7)	39 (46.4)	26 (40.0)	0.405
History of hypertension	15 (68.2)	20 (66.7)	50 (59.5)	33 (50.8)	0.346
History of diabetes	11 (50.0)	18 (60.0)	34 (40.5)	26 (40.0)	0.234
History of myocardial infraction	0 (0.0)	0 (0.0)	1 (1.2)	1 (1.5)	0.861
Family history of CHD	3 (13.6)	4 (13.3)	16 (19.0)	11 (16.9)	0.870
Family history of diabetes	0 (0.0)	2 (6.7)	6 (7.1)	5 (7.7)	0.626
**Lab examination**					
HbA1c (%)	6.74 ± 0.98	6.78 ± 0.81	7.29 ± 1.15	7.25 ± 1.27	0.052
Glucose (mmol/L)	6.03 ± 2.00	6.06 ± 1.80	6.26 ± 6.17	6.40 ± 2.17	0.978
ALB (g/L)	46.05 ± 5.56	44.80 ± 4.86	44.42 ± 3.46	44.79 ± 3.87	0.429
PA (mg/L)	26.44 ± 4.52	25.81 ± 5.15	25.62 ± 3.96	26.36 ± 4.49	0.720
Homocysteine (μmol/L)	18.55 ± 7.61	15.54 ± 3.80	16.45 ± 5.63	16.26 ± 6.56	0.326
Serum amyloid A (mg/L)	7.78 ± 1.47	7.29 ± 1.40	7.61 ± 1.48	7.37 ± 1.45	0.465
**Medical therapy history**					
Aspirin [yes(%)]	21 (95.5)	28 (93.3)	75 (89.3)	46 (70.8)^##^^&&^	0.002
ADP-R blockers	11 (50.0)	17 (56.7)	45 (53.4)	29 (44.6)	0.643
β-R blockers	12 (54.5)	20 (66.7)	56 (66.7)	27 (41.5)^&&^	0.013
ACEI	6 (27.3)	4 (13.3)	12 (14.3)	5 (7.7)	0.138
ARB	3 (13.6)	11 (36.7)	24 (28.6)	13 (20.0)	0.167
Statins	17 (77.3)	24 (80.0)	65 (77.4)	38 (58.5)^&^	0.040

Continuous variables are recorded by mean ± standard deviation; categorical variables are recorded in proportions. A p value of < 0.05 is considered to be statistically significant.

## p < 0.01 vs. 1.4 mmol/L < LDL ≤ 1.8 mmol/L.

& p < 0.05,

&& p < 0.01 vs. 1.8 mmol/L < LDL ≤ 2.6 mmol/L.

For the drugs that were beneficial for prognosis, the application of aspirin, β-blockers, and statins among the four groups was significantly different. The utilization rate of aspirin in the 1.4 mmol/L < LDL-C ≤ 1.8 mmol/L group was higher than that in the LDL-C > 2.6 mmol/L group (*P* < 0.01, Table 1). And the rates of aspirin, β-blockers, and statins in the 1.8 mmol/L < LDL-C ≤ 2.6 mmol/L group were higher than that in the LDL-C > 2.6 mmol/L group (*P* < 0.05, Table 1). No statistical difference was found in the usage of ADP receptor blockers, angiotensin-converting enzyme inhibitors (ACEIs), and angiotensin II receptor blockers (ARBs) among the four groups (*P* > 0.05, Table 1).

Considering the possible impact of sample size on the drug response results, we reclassify patients into the following three groups, namely, the LDL-C ≤ 1.8 mmol/L group, 1.8 mmol/L < LDL-C ≤ 2.6 mmol/L group, and LDL-C > 2.6 mmol/L group. The usage rates of drugs were recalculated (Supplementary Table 1). The utilization rate of aspirin and statins in the LDL-C ≤ 1.8 mmol/L group was higher than that in LDL-C > 2.6 mmol/L group (*P* < 0.05). And the rates of aspirin, β-receptor blockers, and statins in the 1.8 mmol/L < LDL-C ≤ 2.6 mmol/L group were higher than that in the LDL-C > 2.6 mmol/L group (*P* < 0.05). The drug utilization rates were consistent under two grouping methods.

Considering the possible impact of drug utilization on each sex, the rates of drug use for each gender were calculated respectively in 2 classifications (Supplementary Tables 2, 3). The characteristics of drug use were similar in males and females, with decreased rates of aspirin and statins in the higher LDL-C group than that in the lower LDL-C group, which was consistent with the baseline data of the total population.

Baseline characteristics indicated that no difference was found in the risk factors of CHD except for LDL-C levels. Consequently, we question whether the lower LDL-C and higher usage rate of drugs in CHD patients are related to decreased size of coronary lesions.

### CHD patients with different LDL-C levels had the same degree of coronary lesions

**Figure 1:**
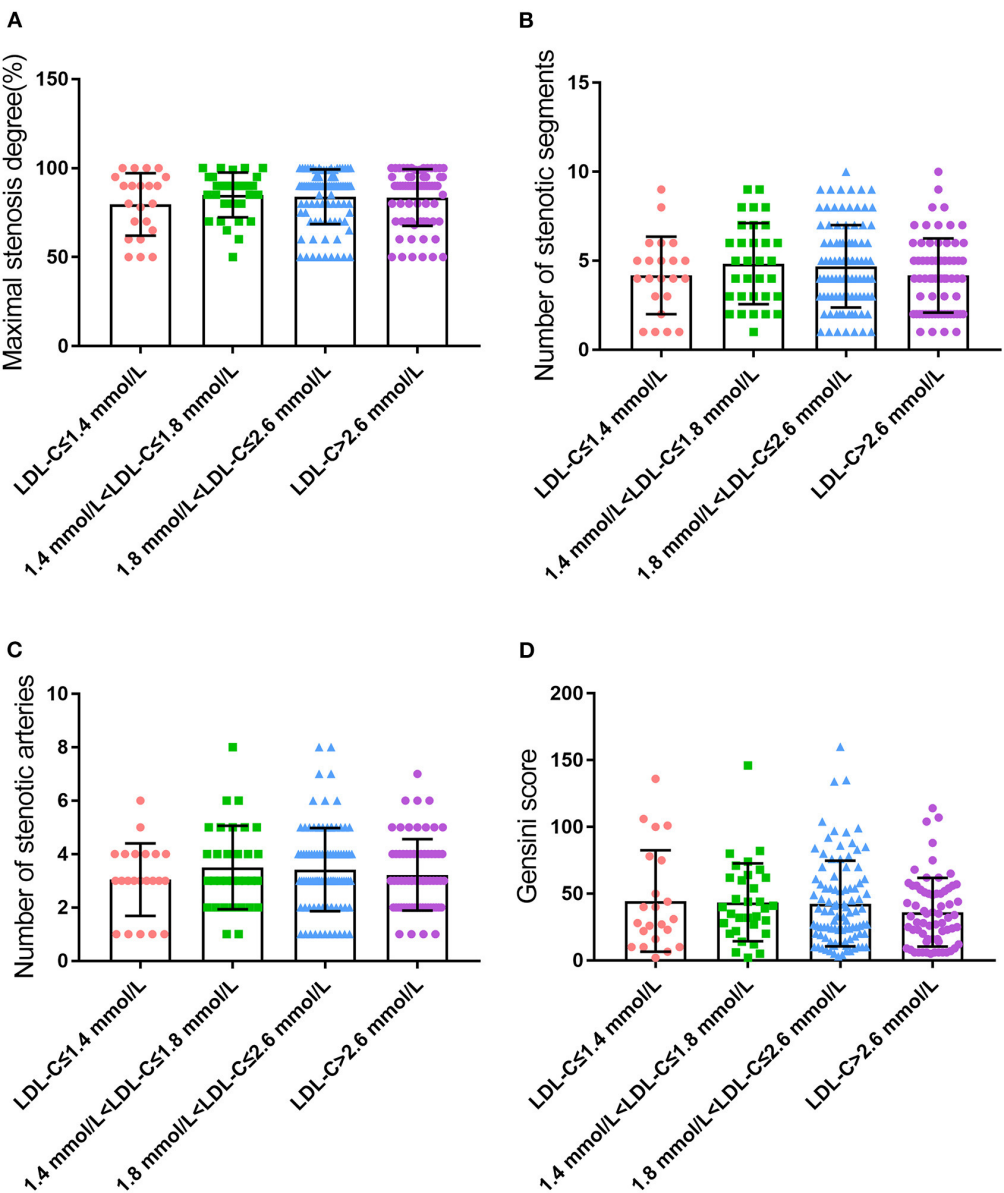
Coronary lesions in CHD patients with different LDL-C levels **(A)**, maximal stenosis degree; **(B)**, number of stenotic segments; **(C)**, number of stenotic arteries; and **(D)**, Gensini score.

Four indicators, maximal stenosis degree, number of stenotic segments, number of stenotic arteries, and Gensini score, were used to assess the severity of coronary lesions. Surprisingly, our results indicated that no statistical difference was found in those four indicators among the four groups with varying LDL-C (*P* > 0.05, Figure 1). The differences in four indicators were also not statistically significant when patients were divided into three groups according to LDL-C levels as described previously (*P* > 0.05, Supplementary Figure 1). This striking result revealed that the degree of coronary lesions in CHD patients was independent of LDL-C level in the case where other factors were completely comparable. However, other contributory factors of coronary lesions except for LDL-C are still unclear.

### β-sheet content of serum protein was negatively correlated with LDL-C level in CHD patients

**Figure 2:**
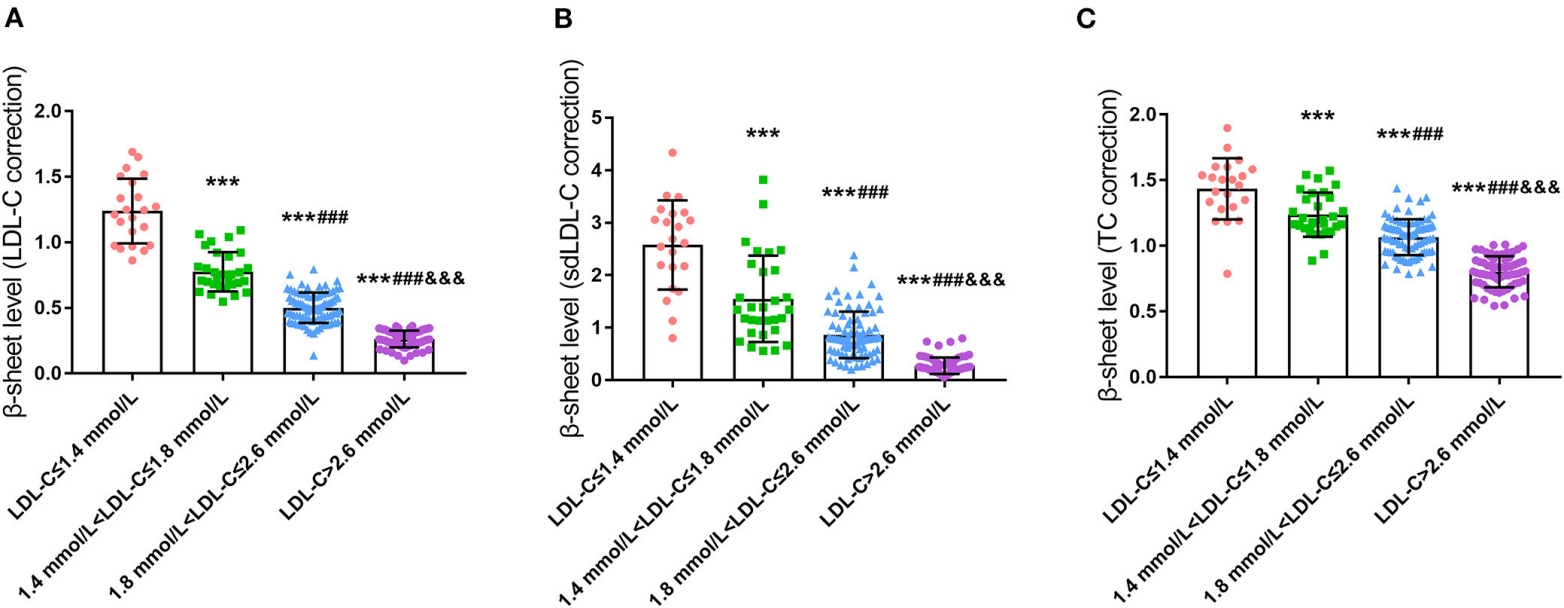
Comparison of serum β-sheet levels in CHD patients with different LDL-C levels **(A)**, LDL-C correction; **(B)**, sdLDL-C correction; **(C)**, TC correction; ^***^p < 0.001 vs. LDL-C ≤ 1.4 mmol/L. ^###^*p* < 0.001 vs. 1.4 mmol/L < LDL ≤ 1.8 mmol/L. ^&&&^*p* < 0.001 vs. 1.8 mmol/L < LDL ≤ 2.6 mmol/L.

Serum ThT fluorescence intensity was utilized to reflect the content of the β-sheet in peripheral blood. To our surprise, the content of the β-sheet was significantly different among groups after correction for LDL-C, small dense LDL-C (sdLDL-C), and total cholesterol (TC), which was consistent under both grouping methods (*P* < 0.001, Figure 2 and Supplementary Figure 2). Further analysis revealed that the β-sheet level was increased significantly and concomitantly with the reduced level of LDL-C.

### Relationship between β-sheet level and coronary lesions: Multiple linear regression analyses

Multivariate linear regression was performed with the Gensini score as the dependent variable, and serum β-sheet content and other risk factors as independent variables. As shown in Table 2, a positive correlation was found between the Gensini score and β-sheet, age, systolic blood pressure, and heart rate. In terms of medical history, β-sheet and smoking, diabetes, hypertension, hyperlipidemia, and family history of CHD were positively correlated with the Gensini score (Table 3). In addition, β-sheet and several important biochemical indicators (HbA1c, TC, triglyceride, LDL-C, and sdLDL-C) were positively correlated with the Gensini score (Table 2). In medical therapy, β-sheet and history of aspirin, ADP receptor antagonists, and ARBs were positively correlated with the Gensini score when all drugs were analyzed (Table 3). In the subgroup analysis, β-sheet and history of hypoglycemic agents (biguanides, glycosidase inhibitors, and insulin), lipid-lowering drugs (statins), and antihypertensive medicines (calcium channel blockers, ARBs, ACEIs, and β-receptor blockers) were positively correlated with the Gensini score (Tables 2, 3).

**Table 2:** Summary of multiple linear regression models for β-sheet level and Gensini score (univariate).

**Independent variable**	**β**	***P***
β-sheet & age	0.075 & 0.900	0.036 & <0.001
β-sheet & SBP	0.093 & 0.883	0.011 & <0.001
β-sheet & heart rate	0.116 & 0.853	0.004 & <0.001
β-sheet & HbA1c	0.129 & 0.825	0.015 & <0.001
β-sheet & TC	0.321 & 0.698	<0.001 & <0.001
β-sheet & TG	0.513 & 0.457	<0.001 & <0.001
β-sheet & LDL-C	0.400 & 0.641	<0.001 & <0.001
β-sheet & sdLDL-C	0.483 & 0.535	<0.001 & <0.001
β-sheet & aspirin	0.162 & 0.375	0.003 & <0.001
β-sheet & statins	0.483 & 0.457	<0.001 & <0.001

&, and.

**Table 3:** Summary of multiple linear regression models for β-sheet level and Gensini score (multivariate).

**Independent variable**	**β**	***P***
**Total impact factors**		
Age	0.123	0.004
SBP	0.032	<0.001
Smoking	0.315	0.005
HbA1c	0.023	<0.001
LDL-C	0.060	0.030
Aspirin	0.096	0.020
Statins	0.110	0.027
**Medical history**		
β-sheet	0.391	<0.001
Smoking	0.227	<0.001
History of diabetes	0.150	0.001
History of hypertension	0.240	<0.001
History of hyperlipidemia	0.116	0.001
Family history of CHD	0.073	0.046
**Medical therapy**		
β-sheet	0.162	0.003
Aspirin	0.375	<0.001
ADP receptor antagonists	0.105	0.016
ARB	0.076	0.029
**Hypoglycemic agents**		
β-sheet	0.506	<0.001
Biguanide	0.198	0.009
Glycosidase inhibitor	0.133	0.034
Insulin	0.163	0.007
**Antihypertensive drugs**		
β-sheet	0.482	<0.001
CCB	0.126	0.001
ARB	0.088	0.033
ACEI	0.115	0.002
β-receptor blockers	0.298	<0.001

The results mentioned above suggested that the β-sheet level was an independent risk factor for coronary damage in CHD patients. Serum β-sheet level was positively correlated with coronary lesion when risk factors such as age, smoking history, blood pressure, blood glucose, and LDL-C were controlled.

## Discussion

In this study, the baseline characteristics of CHD patients with different LDL-C levels were exactly comparable. The utilization rate of medicines, such as aspirin, β-blockers, and statins was lower in the higher LDL-C group, which was consistent in the total population and both sexes. Theoretically, LDL-C is a key determinant of coronary lesions when other risk factors are comparable [age, hypertension, smoking, and obesity (22–24)]. Considering the possible impact of sample size, two classifications were applied to the total population with cut-off values of 1.4, 1.8, 2.6, and 1.8, 2.6, respectively. Unexpectedly, groups with different LDL-C revealed similar coronary lesions. This means that there were still residual risks in CHD patients whose LDL-C dropped below 1.8 or even 1.4 mmol/L. It is probable that some elevated risk factors overlap the protective effect of low-level LDL-C and contribute to the consistent coronary damage in four groups.

Atherosclerosis is promoted by LDL-C *via* intimal lipid deposition, foam cell formation, and pro-inflammatory substance release (25). LDL-C is currently recommended as the primary target for CHD treatment despite the controversial values (4). In recent years, the residual risk of CHD apart from LDL-C has become increasingly recognized (26–28). Studies have revealed an increased incidence of cardiovascular events and mortality in CHD patients even though diet therapy reduced total cholesterol by 13% (29), and even after the aggressive use of statins or proprotein convertase subtilisin/kexin type 9 inhibitors, which bring LDL-C down to standard, a subset of the CHD population still suffers from adverse cardiovascular events (30, 31). These studies have revealed that risk factors other than cholesterol of LDL-C might be involved in coronary damage.

Latest studies suggested that atherosclerosis could be attributed to the accumulation of cytotoxic and pro-inflammatory misfolded lipoproteins (32). These effects are analogous to those of β-amyloid and possibly many other misfolded proteins, and represent a degenerative-inflammatory process in the arterial wall (15, 16, 33). The link between β-sheet-rich misfolded proteins and atherosclerosis is gaining attention. Proteins in circulation all contain β-sheet, while the content varies. Therefore, we compared the serum β-sheet level of CHD patients in two classifications. Surprisingly, the content of β-sheet was not equilibrious but dramatically increased with the decline of LDL-C. The β-sheet content was significantly elevated in LDL-C ≤ 1.8 group compared with that in the higher LDL-C level group. Further subgroups showed that β-sheet levels were also elevated in the LDL-C ≤ 1.4 group. As far as we know, this is the first report showing the serum β-sheet levels of CHD patients gradually increased with decreasing LDL-C when coronary lesions were comparable. The β-sheet is involved in coronary damage, while it remains unclear where the elevated β-sheet comes from. Evidence from structural biology will shed new light.

As the cholesterol transporter and structural protein of LDL-C (34), ApoB-100 is demonstrated to be misfolded in atherosclerosis (35). Pentagonal ApoB-100 is composed of alternating α-helix and β-sheet domains (NH2-α1-β1-α2-β2-α3-COOH) (36). ApoB-100 is the most likely source of β-sheet since there is no β-sheet in cholesterol. It has been indicated that ApoB-100 is co-located with β-sheet-formed amyloid proteins and foam cells in coronary plaques (10). A significant increase in β-sheet content and a decline of α-helix have been found in electronegative LDL-C *in vitro* (13). Similar results were also found *in vivo* (11, 37).

**Figure 3:**
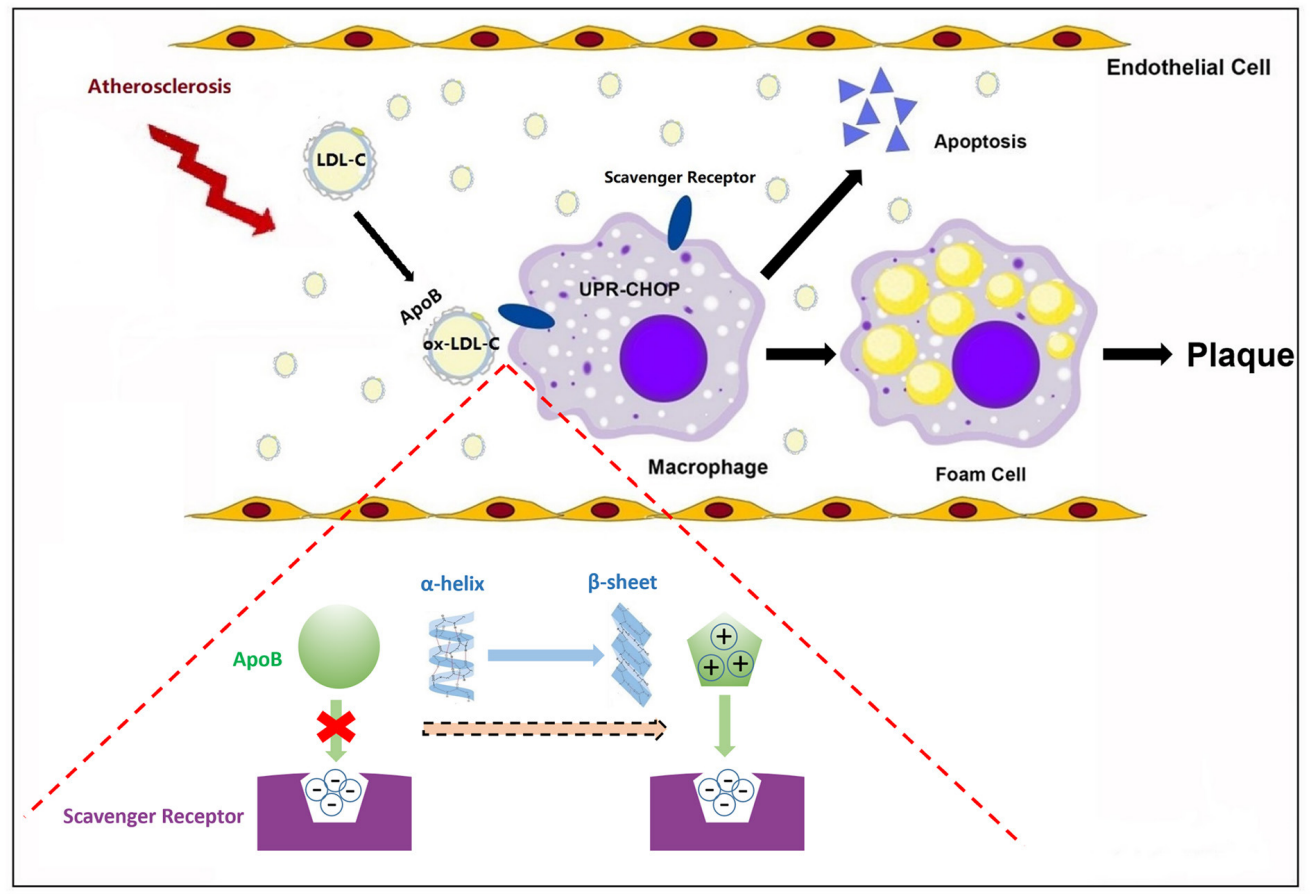
β-sheet conformational change of apolipoproteins in atherosclerosis. In atherosclerosis, the α2 domain of ApoB transforms from α-helix to β-sheet. The β-sheet may not only form a positively charged ligand structural domain but also a structurally complementary spatial structural domain that facilitates the binding of ox-LDL to scavenger receptors, initiating macrophage apoptosis and promoting atherosclerotic plaque formation.

Under physiological conditions, macrophage foaming is not caused by LDL-C due to the negative feedback regulation of cholesterol uptake. The α2 domain of ApoB is prone to transform from α-helix to a more structurally stable β-sheet during LDL-C oxidation, leading to an increased content of β-sheet in ox-LDL-C (12). Lipid peroxidation is the main cause of increased β-sheet in ApoB. The phospholipid C2-site polyunsaturated fatty acids of LDL-C are prone to peroxidation and their products covalently cross-link with lysine residues on ApoB, altering the conformation of ApoB. The β-sheet structure is further stabilized by the rearrangement of the hydrogen bonding pattern triggered by the covalent cross-linking of lysine residues (38). After oxidative modification of LDL-C, which can only be recognized by scavenger receptors, there is no negative feedback regulation of this pathway and oxidized LDL-C is constantly taken up by macrophages, where it accumulates in excess and stimulates macrophages to form foam cells (39). In this process, the β-sheet-rich ApoB may not only form a positively charged ligand structural domain but also a structurally complementary spatial structural domain that facilitates the binding of ox-LDL to the scavenger receptor (40). Endoplasmic reticulum stress in macrophages caused by cholesterol deposition selectively down-regulates cell survival signals via the unfolded protein response-class A scavenger receptor pathway, leading to macrophage apoptosis, atheromatous plaque instability, and rupture (41) (Figure 3). Given that how serum β-sheet might exacerbate the coronary lesions in CHD remains unclear, this assumption remains to be verified jointly by structural biology and molecular biology. In conclusion, β-sheet-rich misfolded proteins are involved in coronary damage. The α2 domain of ApoB transforms from α-helix to β-sheet during cholesterol oxidation and may be largely responsible for the increased content of serum β-sheet in CHD patients. Whether the effect of β-sheet is independent remains obscure.

Serum β-sheet levels differed significantly among four groups with varying LDL-C and comparable risk factors. Multiple linear regression indicates that the effect of β-sheet on coronary lesions was independent of physical examination, medical history, laboratory examination and medication history, and had no interaction with other factors. This preliminary finding demonstrated that coronary damage in CHD patients was increased with elevated serum β-sheet levels when risk factors such as age, smoking history, blood pressure, blood glucose, and LDL-C were controlled.

## Limitations and future directions

The study of β-sheets and misfolded proteins in coronary heart disease is still in its infancy, and many questions remain to be addressed. For example, our study indicated that the percentage of drug use was similar in male and female CHD patients, and the effect of drug use in different genders on coronary lesions was limited in this study. Considering the sample size, these results remain to be confirmed with large cohorts. In addition, how the serum β-sheet exacerbates the coronary lesions in CHD remains to be elucidated jointly by structural biology and molecular biology.

## Conclusion and implications

As far as we know, this is the first report that evaluates the serum β-sheet levels of CHD patients with different LDL-C, and also the first attempt to establish a link between β-sheet and coronary artery damage, which opens a new arena of thought in the future. Despite the limitations, our study has shed light on an unacquainted role of misfolded β-sheets that contribute to the remaining risks of CHD. β-sheets are expected to become a potential target for cardiovascular risk evaluation and a promising target for CHD treatment.

## Data availability statement

The raw data supporting the conclusions of this article will be made available by the authors, without undue reservation.

## Ethics statement

The studies involving human participants were reviewed and approved by Ethics Committee of the Qilu Hospital of Shandong University. The patients/participants provided their written informed consent to participate in this study.

## Author contributions

Y-lL wrote this manuscript. J-yX, BL, and X-dS completed the ThT experiment. Y-lL and Z-jT participated in the data integration and analysis. F-fC and W-wS provided constructive ideas to this manuscript. WZ, MZ, and Z-hW were the designer and reviewer of this work. Z-hW is responsible for the integrity of this study. All authors read and approved the final manuscript.

## Funding

This work was supported by research grants from the National Natural Science Foundation of China (81873534, 81570400, 81470560), the Key research and development program of Shandong Province (2018GSF118002, 2018GSF118017).

## Conflict of interest

The authors declare that the research was conducted in the absence of any commercial or financial relationships that could be construed as a potential conflict of interest.

## Publisher's note

All claims expressed in this article are solely those of the authors and do not necessarily represent those of their affiliated organizations, or those of the publisher, the editors and the reviewers. Any product that may be evaluated in this article, or claim that may be made by its manufacturer, is not guaranteed or endorsed by the publisher.

## Abbreviations

ACEI: angiotensin converting enzyme inhibitors
ALB: albumin
Apo: apolipoprotein
ARB: angiotensin II receptor blockers
BMI: body mass index
CCB: calcium channel blockers
CHD: coronary heart disease
HbA1c: glycosylated hemoglobin
LDL: low-density lipoprotein
LDL-C: low-density lipoprotein-cholesterol
ox-LDL: oxidized low-density lipoprotein
PA: prealbumin
sdLDL-C: small dense low-density lipoprotein cholesterol
TC: total cholesterol
TG: triglyceride
ThT: thioflavin T.
