# Supplementary material for: β-sheets in serum protein are independent risk factors for coronary lesions besides LDL-C in coronary heart disease patients
Source: Front Cardiovasc Med. 2022 Aug 9;9:911358. doi: 10.3389/fcvm.2022.911358 (PMC9395970; doi:10.3389/fcvm.2022.911358)
Supplement: Supplementary file 1 [file Data_Sheet_1.pdf]

Supplementary Material

1 Supplementary Tables

Supplementary Table 1

Medical Therapy History of CHD patients with different LDL-C levels (3 groups)

Characteristic	LDL-C≤1.8 mmol/L （n=52）	1.8 mmol/L <LDL-C≤2.6 mmol/L （n=84）	LDL-C>2.6 mmol/L （n=65）	*P*
Aspirin(yes(%))	49(94.2)	75(89.3)	46(70.8)[*#]	<0.001
ADP-R blockers	28(53.8)	45(53.4)	29(44.6)	0.463
β-R blockers	32(61.5)	56(66.7)	27(41.5)[#]	0.002
ACEI	10(19.2)	12(14.3)	5(7.7)	0.157
ARB	14(26.9)	24(28.6)	13(20.0)	0.445
Statins	41(78.8)	65(77.4)	38(58.5)[*#]	0.009

[*]$p<0.05$ vs. LDL≤1.8 mmol/L. [#]$p<0.05$ vs. 1.8 mmol/L<LDL≤2.6 mmol/L.

Supplementary Table 2

Medical Therapy History of CHD patients by gender (4 groups)

Characteristic	LDL-C≤1.4 mmol/L	1.4 mmol/L <LDL-C≤1.8 mmol/L	1.8 mmol/L <LDL-C≤2.6 mmol/L	LDL-C>2.6 mmol/L	*P*
			Male		
Sample Size	n=14	n=19	n=49	n=39	
Aspirin(yes(%))	14(100)	17(89.5)	40(81.6)	26(66.7)[*#]	0.029
ADP-R blockers	6(42.9)	10(52.6)	23(46.9)	16(41.0)	0.853
β-R blockers	7(50)	13(68.4)	33(67.3)	17(43.6)	0.983
ACEI	4(28.6)	3(15.8)	9(18.4)	3(7.7)	0.272
ARB	1(7.1)	7(36.8)	11(22.4)	6(15.4)	0.148
Statins	9(64.3)	13(68.4)	42(85.7)	21(53.8)[&]	0.012
			Female		
Sample Size	n=8	n=11	n=35	n=26	
Aspirin(yes(%))	7(87.5)	11(100)	35(100)	20(76.9)[#&]	0.011
ADP-R blockers	5(62.5)	7(63.6)	22(62.9)	13(50)	0.748
β-R blockers	5(62.5)	7(63.6)	23(65.7)	10(38.5)	0.173
ACEI	2(25.0)	1(9.1)	3(8.6)	2(7.7)	0.524
ARB	2(25.0)	3(27.3)	13(37.1)	7(26.9)	0.797
Statins	8(100)	11(100)	23(65.7)	17(65.3)[*#]	0.031

[*]$p<0.05$ vs. LDL≤1.4 mmol/L. [#]$p<0.05$ vs. 1.4 mmol/L<LDL≤1.8 mmol/L. [&]$p<0.05$ vs. 1.8 mmol/L<LDL≤2.6 mmol/L.

Supplementary Table 3

Medical Therapy History of CHD patients by gender (3 groups)

Characteristic	LDL-C≤1.8 mmol/L	1.8 mmol/L <LDL-C≤2.6 mmol/L	LDL-C>2.6 mmol/L	*P*
Male				
Sample Size	n=33	n=49	n=39	
Aspirin(yes(%))	31(93.9)	40(81.6)	26(66.7)[#]	0.015
ADP-R blockers	16(48.5)	23(46.9)	16(41.0)	0.789
β-R blockers	20(60.6)	33(67.3)	17(43.6)	0.076
ACEI	7(21.2)	9(18.4)	3(7.7)	0.233
ARB	8(24.2)	11(22.4)	6(15.4)	0.602
Statins	22(66.7)	42(85.7)	21(53.8)[#]	0.004
Female				
Sample Size	n=19	n=35	n=26	
Aspirin(yes(%))	18(94.7)	35(100)	20(76.9)[*#]	0.006
ADP-R blockers	12(63.2)	22(62.9)	13(50)	0.363
β-R blockers	12(63.2)	23(65.7)	10(38.5)	0.083
ACEI	3(15.8)	3(8.6)	2(7.7)	0.625
ARB	5(26.3)	13(37.1)	7(26.9)	0.604
Statins	19(100)	23(65.7)	17(65.3)[*#]	0.012

[*] $p<0.05$ vs. LDL≤1.8 mmol/L. [#] $p<0.05$ vs. 1.8 mmol/L<LDL≤2.6 mmol/L.

2 Supplementary Figures

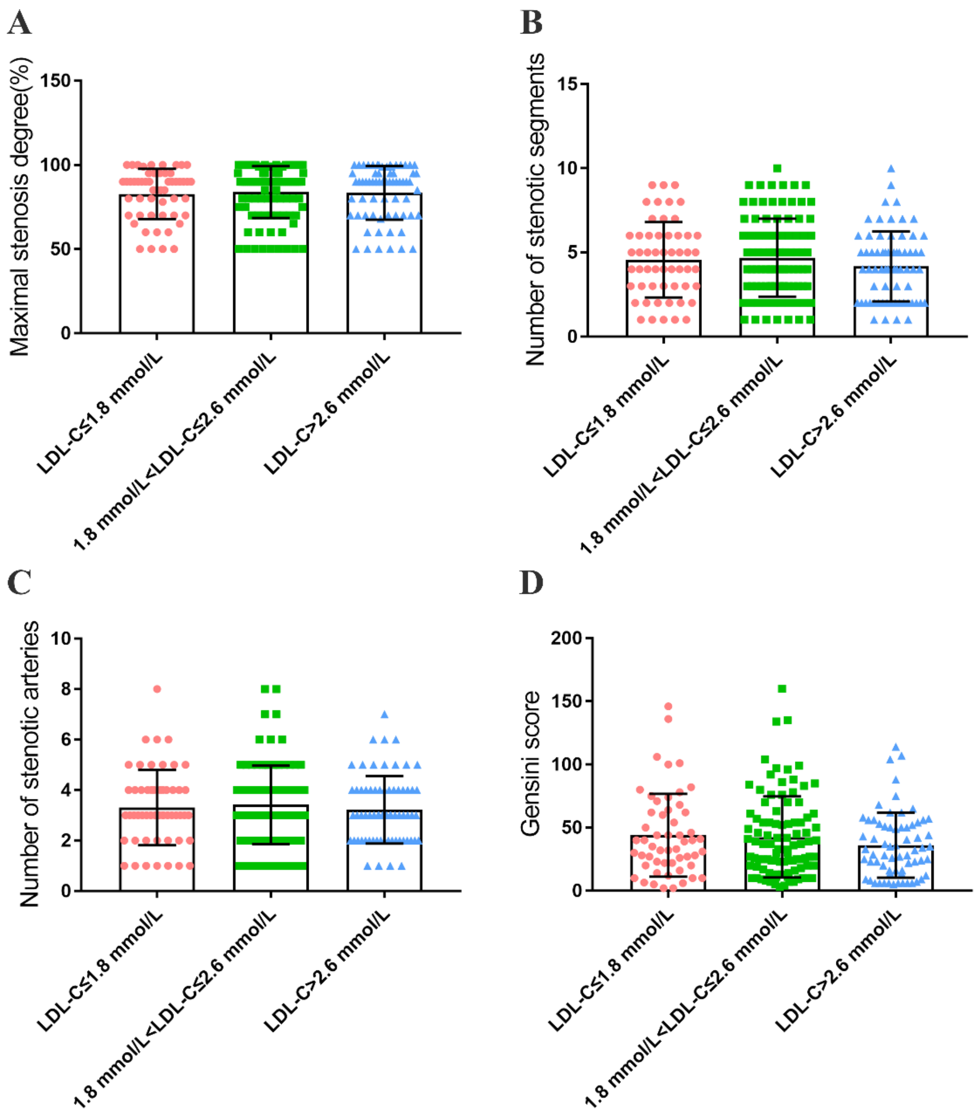

Supplementary Figure 1. Coronary lesions in CHD patients with different LDL-C levels (3 groups) A: maximal stenosis degree, B: number of stenotic segments, C: number of stenotic arteries, D: Gensini score.

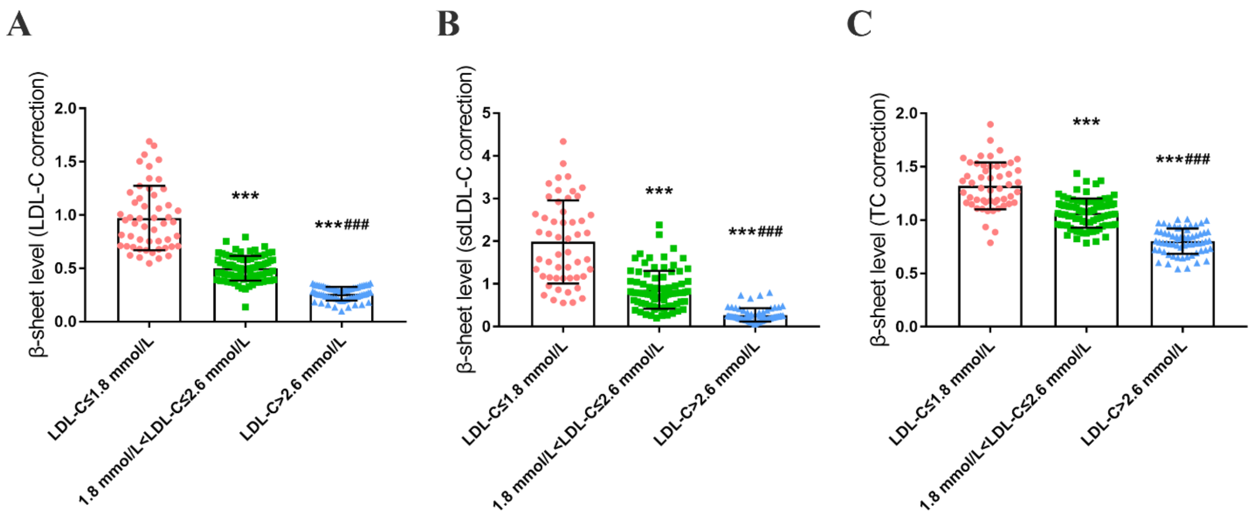

Supplementary Figure 2. Comparison of serum β-sheet level in CHD patients with different LDL-C levels (3 groups). A: LDL-C correction, B: sdLDL-C correction, C: TC correction; $^{***}p<0.001$ vs. LDL-C⩽1.8 mmol/L. $^{###}p<0.001$ vs. 1.8 mmol/L<LDL ⩽ 2.6 mmol/L.
